# A Multiple Kernel Learning Model Based on *p*-Norm

**DOI:** 10.1155/2018/1018789

**Published:** 2018-01-23

**Authors:** Jinshan Qi, Xun Liang, Rui Xu

**Affiliations:** ^1^School of Information, Renmin University of China, Beijing 100872, China; ^2^School of Computer Science and Technology, Huaiyin Normal University, Huai'an, Jiangsu 223300, China

## Abstract

By utilizing kernel functions, support vector machines (SVMs) successfully solve the linearly inseparable problems. Subsequently, its applicable areas have been greatly extended. Using multiple kernels (MKs) to improve the SVM classification accuracy has been a hot topic in the SVM research society for several years. However, most MK learning (MKL) methods employ *L*_1_-norm constraint on the kernel combination weights, which forms a sparse yet nonsmooth solution for the kernel weights. Alternatively, the *L*_*p*_-norm constraint on the kernel weights keeps all information in the base kernels. Nonetheless, the solution of *L*_*p*_-norm constraint MKL is nonsparse and sensitive to the noise. Recently, some scholars presented an efficient sparse generalized MKL (*L*_1_- and *L*_2_-norms based GMKL) method, in which *L*_1_  *L*_2_ established an elastic constraint on the kernel weights. In this paper, we further extend the GMKL to a more generalized MKL method based on the *p*-norm, by joining *L*_1_- and *L*_*p*_-norms. Consequently, the *L*_1_- and *L*_2_-norms based GMKL is a special case in our method when *p* = 2. Experiments demonstrated that our *L*_1_- and *L*_*p*_-norms based MKL offers a higher accuracy than the *L*_1_- and *L*_2_-norms based GMKL in the classification, while keeping the properties of the *L*_1_- and *L*_2_-norms based on GMKL.

## 1. Introduction of MKL

The support vector machine (SVM) is a classification and regression tool based on the statistical machine learning [1]. By utilizing the kernel function, the SVM transfers the data into a high dimension space, builds an optimal separating hyperplane, and consequently solves the nonlinear problem. In solving an SVM problem, it is critical to choose an adequate kernel function. The widely used kernel functions are the radial basis functions and polynomial functions. To select an effective kernel function is very important, and different kernels and parameters produce different classification and regression results. In our paper, we try to use the features of different kernels and improve the classification accuracy of SVM.

The multiple kernel learning (MKL) model [2] is a flexible learning model. In the recent research, the MK learning (MKL) can obtain higher classification accuracy than the sole one. As the MKL uses different combinations of kernel functions and has larger flexibility, its performance is normally better. Constructing the MK model, in fact, is the process of seeking the combination of *M* kernels to get the best classification accuracy. Thus, in the MK framework, to seek the weights of the different kernels is the big problem for MKL [3, 4]. The simplest form of MKL is *L*_1_ norm [5]. The *L*_1_-norm MKL finds the kernel weight in a simplex form and thus yields a sparse solution [6, 7]. The sparsity of selected kernels is helpful in identifying an appropriate combination of data sources or subsets with different features in real world applications. However, the method may discard useful information and thus result in a suboptimal generalization.

Alternatively, the *L*_2_-norm MKL was proposed by another group of researchers, and it improves *L*_1_-norm MKL in some scenarios. Unfortunately, the solution of *L*_2_-norm MKL is nonsparse, which means it uses all kernels in the forecasting stage. Also, the *L*_2_-norm MKL is sensitive to noise. Additionally, when there exist noisy data in the training set, the classification accuracy would be greatly decreased. Furthermore, it suffers poor interpretation and can lead to high computational and storage cost, too.

Thus, there is research intending to combine the *L*_1_-norm MKL and *L*_2_-norm MKL. The algorithm is called the generalized MKL (GMKL) [8], which combines both advantages of *L*_1_- and *L*_2_-norms and is able to have a higher accuracy in classifications. Nonetheless, the GMKL algorithm is just specialized in the combination of the sparse MKL method and the nonsparse kernel learning method, *L*_2_-norm MKL. The research made a contribution to the merging of the *L*_1_- and *L*_2_-norm MKL, and the GMKL in a general model [9]. In this paper, we extend the algorithm in a more general form, which combines the sparse MKL and* all *nonsparse MKL algorithms. Thus, we would like to generalize the *L*_2_-norm MKL to the *L*_*p*_-norm.

In our paper, we combine *L*_1_- and *L*_*p*_-norms [10], by extending the constraint of kernels as *v*∑_*j*=1_^*M*^*u*_*j*_ + (1 − *v*)∑_*j*=1_^*M*^*u*_*j*_^*p*^ ≤ 1. We call our algorithm MKL based on *p*-norm (MKL-BP). In particular, when *p* = 2 the MKL-BP algorithm will be degenerated into the GMKL algorithm. In our experiments, when *p* → *∞*, the accuracy of our algorithm tends to be stable and is higher than the results with *p* = 2. Meantime, compared with the *L*_1_- and *L*_*p*_-norm MKL method, the MKL-BP shows the higher accuracy in the classifications too. The advantage of using *L*_*p*_ norms is that more flexibility can be achieved during the experiments. As *p* changes, the generalization and precision vary accordingly.

The paper is organized as follows: Section 2 describes in detail the MKL-BP model.  Section 3 analyzes and verifies the relevant definitions and theorems of MKL-BP model. The implementation solution of MKL-BP model is described in Section 4.  Section 5 uses the MKL-BP model to carry out experiments on the UCI datasets and compares its accuracy, running time, and so on with those of other MKL models.  Section 6 concludes this research with directions for future work.

## 2. Base Framework of MKL-BP

Based on the statistics machine learning in the classification problem, we can get the general model below:

(1) $$\begin{array}{c} f=arg\min Cemp\left(\;f\;\right)+Ω\left(\;f\;\right). \end{array}$$

The smallest empirical risk is 1/*N*∑_*m*=1_^*N*^*R*(*f*(*x*_*m*_), *y*_*m*_), while the smallest regulation risk is Ω(*f*) = 1/2‖*w*‖^2^. The parameter *C* is a presetting constant, used for balancing the empirical and regulation risks.

In the *C*-SVM, the model could be shown as

(2) minw,b⁡  12w2+C∑m=1Nξms.t. yiwTϕxm+b≥1−ξm,m=1,…,N,  ξm≥0.

By optimizing problem (2), the classifier could be shown as

(3) $$\begin{array}{c} f\left(\;x\;\right)=w^{T}\phi \left(\;x\;\right)+b,\;w\in R^{d\left(H\right)},\;\;b\in R. \end{array}$$

Using the Langrage function and kernel *K*(*x*_*m*_, *x*_*n*_) ≤ *ϕ*(*x*_*m*_), *ϕ*(*x*_*n*_) > 0, we could get the dual form of problem (2):

(4) maxα⁡  ∑m=1Nαm−∑m=1N∑n=1NαmαnKxm,xns.t. ∑m=1Nαm=0,0≤αm≤C.

Problem (4) is a simplest form of SVM. In the MKL model, kernel *K* is combined with a series of kernels linearly. The kernel *K* is shown as

(5) $$\begin{array}{c} K=\overset{M}{\underset{j=1}{\sum }}u_{j}K_{j}. \end{array}$$

In (5), *u*_*j*_ refers to the weight of kernel *K*_*j*_, and *M* refers to the number of kernels. By using (5) and replacing *K*(*x*_*m*_, *x*_*n*_) in (4), we can get the standard form of MKL:

(6) minu∈A⁡ maxα⁡  ∑m=1Nαm−∑m=1N∑n=1Nαmαn∑j=1MujKjxm,xns.t. ∑m=1Nαm=0,0≤αm≤C,

where *u* = (*u*_1_,…,*u*_*j*_)^*T*^ and *A* refers to the constraint domain of *u*. In the MKL model, the simplest domain is the *L*_1_-norm MKL, where *A* = {*u*_*j*_∣*u*_*j*_ ≥ 0, ∑_*j*=1_^*M*^*u*_*j*_ ≤ 1}. The research shows that in the *L*_2_- and *L*_*p*_-norm MKLs, where *A* = {*u*_*j*_∣*u*_*j*_ ≥ 0, ∑_*j*=1_^*M*^*u*_*j*_^*p*^ ≤ 1}, there is better classification character in some aspects.

The research combined the *L*_1_- and *L*_2_-norm MKLs, and the GMKL model. The paper showed that the novel model keeps the sparsity of the *L*_1_-norm MKL and the classification accuracy does not decrease when facing the noisy data. Domain *A* in the GMKL model is {*u*_*j*_∣*u*_*j*_ ≥ 0, *v*∑_*j*=1_^*M*^*u*_*j*_ + (1 − *v*)∑_*j*=1_^*M*^*u*_*j*_^2^ ≤ 1}. The setting constant *v* is used to balance the *L*_1_- and *L*_2_-norm MKLs, and 0 ≤ *v* ≤ 1. The experiments showed that when *v* = 0.5, the model gets the best classification accuracy.

However, the paper just specialized the sparse and nonsparse MKL models. In this paper, we would like to generalize the model. Concretely, we generalize domain *A* as {*u*_*j*_∣*u*_*j*_ ≥ 0, *v*∑_*j*=1_^*M*^*u*_*j*_ + (1 − *v*)∑_*j*=1_^*M*^*u*_*j*_^*p*^ ≤ 1}. We called our model the MKL based on *p*-norm (MKL-BP).

We would like to bring the character of our model in the next paragraph, where we will show the model keeping the character of GMKL. Then we give the algorithm of the model to solve the high dimensional constraint problem. We would make some simulation experiments to show the classification accuracy, running time, and used kernel of our model, compared with different models.

## 3. Theorem of MKL-BP

**Theorem 1 .**

Not all the kernels are selected in the MKL-BP model, and *u*_*j*_ of the selected kernels are unique.

**Proof**

By fixing *α* = (*α*_1_,…,*α*_*i*_)^*T*^ as *α*^*∗*^, we could easily know that the optimizing result of *u* in (6) would be irrelevant to *α*^*∗*^. We use the Langrage function and get

(7) Lu=∑m=1Nαm∗−∑m=1N∑n=1Nαm∗αn∗∑j=1MujKjxm,xn+λv∑j=1Muj+1−v∑j=1Mujp.

By trying to get the partial derivatives of *u*_*j*_, we get that

(8) ∂L∂uj=−∑m=1N∑n=1Nαm∗αn∗Kjxm,xn+λv+p1−vujp−1.

By setting ∂*L*/∂*u*_*j*_ = 0, we get *u*_*j*_:

(9) uj=1p1−v1λ∑m=1N∑n=1Nαm∗αn∗Kjxm,xn−vp−1.

Considering when *u*_*j*_ in (9) is below zero, we set *u*_*j*_ as

(10) uj=1p1−v1λ∑m=1N∑n=1Nαm∗αn∗Kjxm,xn−vp−1,0.

From (10), we could easily find that when (1/*λ*)∑_*m*=1_^*N*^∑_*n*=1_^*N*^*α*_*m*_^*∗*^*α*_*n*_^*∗*^*K*_*j*_(*x*_*m*_, *x*_*n*_) < *v*, we get *u*_*j*_ = 0. So not all kernels would be selected in the model when 0 < *v* < 1. Thus, our model successfully selects the useful kernels in optimization. Also, from (10), the optimization result of *u*_*j*_ is unique in our model.

Specially, when *v* = 0, the algorithm is degenerated into the *L*_*p*_-norm MKL, and we get

(11) $$\begin{array}{c} u_{j}=\sqrt[{p-1}]{\frac{1}{p\lambda }\overset{N}{\underset{m=1}{\sum }}\overset{N}{\underset{n=1}{\sum }}\alpha_{m}^{*}\alpha_{n}^{*}K_{j}\left(x_{m},x_{n}\right)}. \end{array}$$

We find that all *u*_*j*_ > 0, which indicates that all kernels are selected in the *L*_*p*_-norm MKL, so it would not discard useful kernels in the optimization. However, the model would not get high accuracy in prediction when faced with noisy data. Also in that scenario, the model may cause higher computational complexity.

**Definition 2 (similar kernel).**

With the optimization of (4) and *α*^*∗*^, if the selected kernels *K*_*j*_ and *K*_*q*_ correspond to the formula below, we call them similar kernels:

(12) ∑m=1N∑n=1Nαm∗αn∗Kjxm,xn−∑m=1N∑n=1Nαm∗αn∗Kqxm,xn≤1.

**Theorem 3 .**

Similar kernels would get the same kernel weights *u*_*j*_ when *p* approaches the limit.

**Proof**

We calculate |*u*_*j*_ − *u*_*q*_| as below:

(13) uj−uq=1p1−v1λ∑m=1N∑n=1Nαm∗αn∗Kjxm,xn−vp−1−1p1−v1λ∑m=1N∑n=1Nαm∗αn∗Kqxm,xn−vp−1≤1p1−v1λ∑m=1N∑n=1Nαm∗αn∗Kjxm,xn−v−1p1−v1λ∑m=1N∑n=1Nαm∗αn∗Kqxm,xn−v≤1pλ1−v.

When *p* approaches to the limit, |*u*_*j*_ − *u*_*q*_| → 0.  Theorem 3 indicates that when *p* approaches the limit, *u*_*j*_ among different kernels would be very small, and thus the classification accuracy does not change.

## 4. Solution of MKL-BP

Although we have presented the MKL-BP model, it is still hard to optimize problem (6). Problem (6) is quadratic programming with a high dimension constraint. In the GMKL algorithm, [11] used the level method to solve the problem. However, in our model, the constraint is *p*-dimensional and the method in [11] does not work. So, we resort to the Taylor expansion method to solve the problem approximately.

We use the coordinate decreasing method to solve the problem in the iteration; we fix *u* or *α*, then solve the subproblem, and finally update *u* or *α*.

**Process 1.**

Update *α* by fixing* u*. At the first time, *u*_*j*_ is initialed as the approximate solution of *vu*_*j*_ + (1 − *v*)*u*_*j*_^*p*^ = 1/*M*; (6) turns to a standard SVM problem below:

(14) maxα⁡  ∑m=1Nαm−∑m=1N∑n=1Nαmαn∑j=1MujtKjxm,xn.s.t. ∑m=1Nαm=0,0≤αm≤C.

Number *t* refers to the iteration time of algorithm. We employ the SMO algorithm to solve this standard problem.

**Process 2.**

Update *u* by fixing *α*; (6) turns to quadratic programming with a high dimensional constraint. Then use the Taylor expansion to decrease the dimension:

(15) up≈utp+putp−1u−ut+pp−1utp−2u−ut22=pp−12utp−2u2+2p−p2utp−1u+p2−3p+22utp.

By using the transformation in (15), the constraint turns to

(16) v∑j=1Muj+1−v∑j=1Mujp=∑j=1M1−vpp−12uj,tp−2uj2+∑j=1Mv+1−v2p−p2uj,tp−1uj+∑j=1Mp2−3p+22uj,tp.

Now with the Taylor expansion, we successfully changed the high dimensional constraint to a quadratic constraint. Next, we use the level method and CVX toolbox as the GMKL to solve the problem in Process 2. CVX toolbox is a useful MATLAB toolbox in solving many mathematic problems.

**Process 3.**

Update *u* or *α* until the stop criterion is satisfied. The stop criterion is that the program has reached the iteration time or the changes of the objective function have reached the threshold.

We could find that when *p* > 2, we successfully changed the problem to the GMKL, so the complexity is the same as that of GMKL. And according to [8], the complexity of GMKL is *O*(*δ*^−2^), when *δ* is the threshold of solution.

## 5. Experiments

In this section we use the UCI data to evaluate the classification accuracies in different algorithms.

We evaluate the following algorithm:

*(1) Ave-Kernel*. We use a base combination of the kernels. The weights of base combination of kernels are *u* = 1/*M*. We use the standard SVM solver to solve the Ave-kernel.

*(2) Simple-MKL*. It is a traditional *L*_1_-MKL model, which is a useful comparison algorithm in many papers.

*(3)*  *L*_*p*_*-MKL*. The constraint of the kernel weight is ‖*u*‖_*p*_ ≤ 1; in our paper we set *v* = 0 as *L*_*p*_-MKL.

*(4) GMKL*. The constraint of kernel weights is {*u*_*j*_∣*u*_*j*_ ≥ 0, *v*∑_*j*=1_^*M*^*u*_*j*_ + (1 − *v*)∑_*j*=1_^*M*^*u*_*j*_^2^ ≤ 1}, and in our paper, we set *p* = 2 as the GMKL.

To be consistent with the past work, all the solvers of the SVM QP are from the LibSVM QP solver. For updating and solving kernel weights, we use the CVX toolbox.

For the SVM parameter *C*, we set it as 100. For the MKL-BP algorithm in our paper, the parameter settings are as below:

The setting of parameter *p* is {2, 3, 4, 5, 6, 7, 8, 16, 32, 64}. When *p* = 2, the algorithm is degenerated to the GMKL. The setting of the parameter *v* is 0.5 as the MKL-BP.

We will use the UCI database to analyze our MKL-BP algorithm; the experiment used 5 UCI datasets. The format of the datasets is given in Table 1.

The setting of kernels is shown as below.

*(1) Gaussian Kernel*. *K*(*x*_*i*_, *x*_*j*_) = *e*^−‖*x*_*i*_ − *x*_*j*_‖/*σ*^2^^. We use 10 parameters {2^−3^,…, 2^6^}.

*(2) Polynomial Kernel*. *K*(*x*_*i*_, *x*_*j*_) = (*x*_*i*_^*T*^*x*_*j*_ + 1)^*d*^. The parameters are {1,2, 3}.

The Gaussian kernel and polynomial kernel are the most popular kernels in SVM, combining them in the same model could combine their character in classification. We imitate the simple-MKL and GMKL algorithm, to normalize the kernel matrix to one unit and we construct 13(*d* + 1) kernels (*d* represents the dimension of data, and the number 13 is the total number of Gaussian kernel and polynomial kernel). We randomly divided the data into two groups. One group with 50% is used for training, and the other group with 50% is for testing. We test the datasets for 50 times to get the same effects of the cross-validation. For every UCI data we run the experiments for 5 times and count the average accuracy of the experiments.

**Figure 1:**
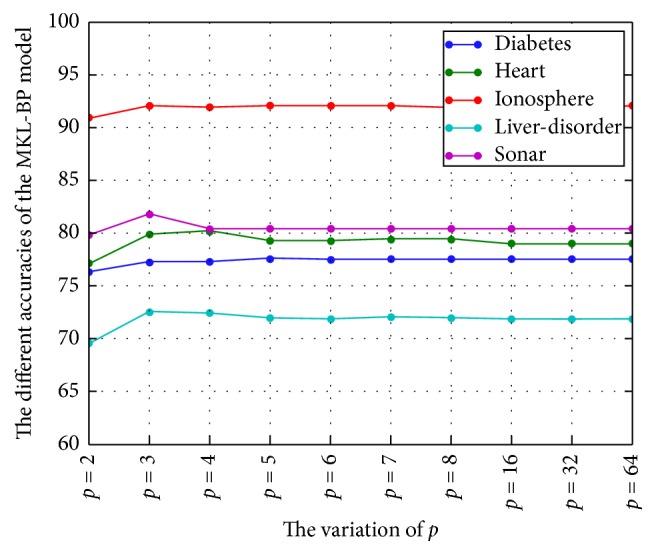
The variation of *p* leads to the different accuracies of the MKL-BP model.

**Figure 2:**
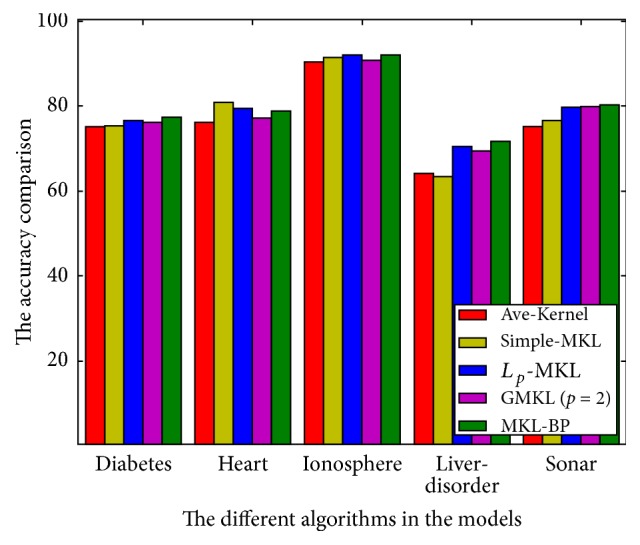
The accuracy comparison of different algorithms.

**Figure 3:**
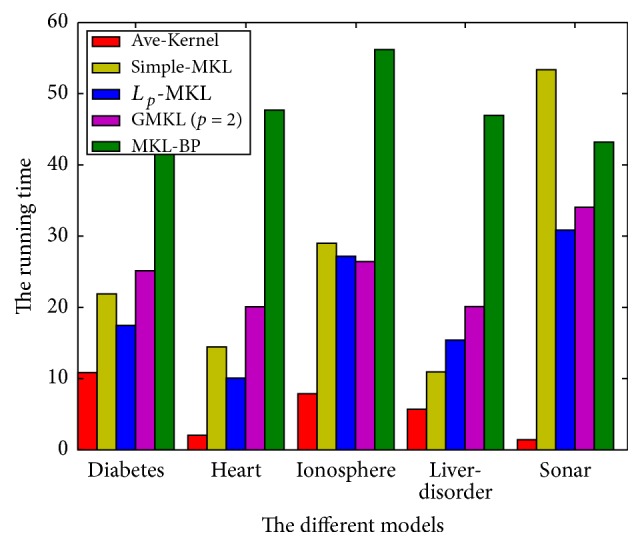
The running time of different models.

**Figure 4:**
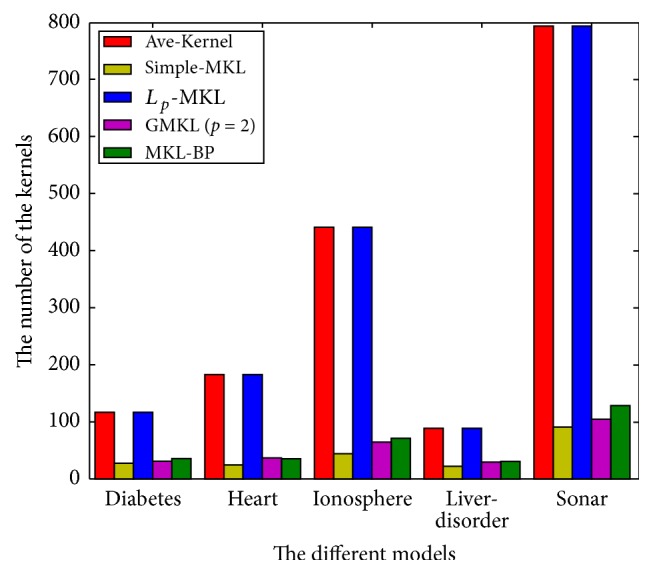
The number of the kernels used in the different models.

(1) The variation of *p* leads to the accuracy of the algorithm: Table 2 shows that when *p* > 2, the accuracy of the MKL-BP model increases in a small scale. Compared with *p* = 2 the, accuracy increases by 1.21%, 1.81%, 1.11%, 2.27%, and 0.58%, respectively. However, from Figure 1 we found that as *p* varies, the accuracy does not change in a large scale.

(2) Compared with other GMKL accuracies, from Table 3 we found that when *p* → *∞*, the MKL-BP model gets better classification accuracy. We discovered that besides the heart data, the MKL-BP model shows the highest accuracy, and the GMKL model (the MKL-BP model when *p* = 2), simple-MKL model, and *L*_*p*_-MKL model all have similar accuracy a little smaller than MKL-BP, while Ave-kernel model reflects the smallest accuracy. The accuracy comparison of different algorithms is also shown in Figure 2.

(3) Compared with other MKL models' running time, Table 4 demonstrates that the Ave-kernel model shows the highest speed, for it only needs to calculate the SVM problem. The running time of *L*_*p*_-MKL model is a bit faster than the simple-MKL model and the GMKL model, and the simple-MKL model and the GMKL model show similar running time. However, the running time of MKL-BP model is the longest. The reason is that by using the Taylor expansion to calculate *u*_*j*,(*t*)_^*p*^, it needs slightly more time. So how to improve the running speed of our algorithm is a problem which needs to be solved in the future research. In order to compare the results clearly, Figure 3 shows the running time of different models.

(4) The variation of number of selected kernel functions is shown in Table 5: the Ave-kernel model and *L*_*p*_-MKL model select all the kernel functions, while simple-MKL model, GMKL model, and MKL-BP model only select a portion of the kernels, which indicates that the MKL-BP keeps the sparsity for the kernel selecting of *L*_1_-MKL model and GMKL model. We discovered that MKL-BP model and the GMKL model select more kernels than the simple-MKL. The reason is that the simple-MKL may discard some useful kernels while the MKL-BP model retains these kernels similarly to the GMKL model.  Figure 4 shows the comparison of the number of the kernels used in the different models.

(5) We could find that when *p* > 2, the accuracy of the MKL-BP model increases in a small scale. However, the accuracy of MKL-BP is not linear to the *p*. For example, the best classification of* heart* is *p* = 4. Therefore, when *p* changes, the advantage of MKL-BP is that we could get higher accuracy than GMKL, but the disadvantage of MKL-BP is that we could not ensure the optimal *p* for the model.

In summary, multiple kernels improve generalization and precision performance in all the experiments, and the running speed of our model is also very fast.

## 6. Conclusion

In our paper we presented a novel MKL model, MKL-BP model, based on the *p*-norm. The model combines *L*_1_-MKL model and *L*_*p*_-MKL model, which generalizes the GMKL model with *p* = 2 to our *p* ≥ 2. The MKL-BP model keeps the sparsity of *L*_1_-MKL model and GMKL model, which only selects useful kernels and makes relatively higher classification accuracy when faced with the noisy data. We use the Taylor expansion to optimize the problem.

From the experiments we found, compared with other MKL models, our MKL-BP model obtains a higher classification accuracy than other models and the kernels selected are much fewer than Ave-kernel model and *L*_*p*_-MKL model. Nevertheless, how to increase classification speed of MKL-BP model is still a problem which we need to solve in the future research.

In the future work, the convergence rates in the experiments may be improved with combining coordinate decreasing method [12].

## Figures and Tables

**Table 1:** Datasets, where Number means the number of the data in the datasets, and Dim means the character of the datasets.

Data name	Number	Dim
Diabetes	768	9
Heart	270	13
Ionosphere	351	33
Liver-disorders	345	6
Sonar	208	60

**Table 2:** The variation of *p* with regard to the different accuracies of the MKL-Bp model.

	*p* = 2	*p* = 3	*p* = 4	*p* = 5	*p* = 6	*p* = 7	*p* = 8	*p* = 16	*p* = 32	*p* = 64
Diabetes	76.2917	77.1875	77.2396	77.5	77.4479	77.5	77.5	77.5	77.5	77.5
Heart	77.1481	79.8519	80.1481	79.2593	79.2593	79.4074	79.4074	78.963	78.963	78.963
Ionosphere	90.9318	92.0455	91.9318	92.0455	92.0455	92.0455	91.9318	92.0455	92.0455	92.0455
Liver-disorder	69.5202	72.4855	72.3699	71.9075	71.7919	72.0231	71.9075	71.7919	71.7919	71.7919
Sonar	79.8077	81.7308	80.3846	80.3846	80.3846	80.3846	80.3846	80.3846	80.3846	80.3846

**Table 3:** The accuracy comparison of different algorithms (the numbers in the brackets are the ranks of different algorithms: 1 means the highest rank in the five models, and 5 means the lowest rank in the models).

	Ave-Kernel	Simple-MKL	*L*_*p*_-MKL	GMKL (*p* = 2)	MKL-BP
Diabetes	75.1224 (5)	75.44 (4)	76.5625 (2)	76.2917 (3)	77.5 (1)
Heart	76.1523 (5)	80.98 (1)	79.4074 (2)	77.1481 (4)	78.963 (3)
Ionosphere	90.5432 (5)	91.48 (3)	92.0455 (1)	90.9318 (4)	92.0455 (1)
Liver-disorder	64.2538 (4)	63.35 (5)	70.4855 (2)	69.5202 (3)	71.7919 (1)
Sonar	75.1146 (5)	76.71 (4)	79.7063 (3)	79.8077 (2)	80.3846 (1)

Average rank	4.8	3.4	2	3.2	1.4

**Table 4:** The running time of different models (the numbers in the brackets are the ranks of different models, and the unit in the table is second(s)).

	Ave-Kernel	Simple-MKL	*L*_*p*_-MKL	GMKL (*p* = 2)	MKL-BP
Diabetes	10.75 (1)	21.83 (3)	17.46 (2)	25.09 (4)	43.44 (5)
Heart	1.96 (1)	14.44 (3)	10.06 (2)	20.08 (4)	47.63 (5)
Ionosphere	7.81 (1)	28.95 (4)	27.14 (3)	26.38 (2)	56.11 (5)
Liver-disorder	5.69 (1)	10.95 (2)	15.30 (3)	20.02 (4)	46.94 (5)
Sonar	1.37 (1)	53.41 (5)	30.76 (2)	33.99 (3)	43.14 (4)

Average rank	1	3.4	2.4	3.4	4.8

**Table 5:** The number of the kernels used in the different models.

	Ave-Kernel	Simple-MKL	*L*_*p*_-MKL	GMKL (*p* = 2)	MKL-BP
Diabetes	117	27	117	30	35.8
Heart	182	25	182	36	35.4
Ionosphere	442	45	442	64.6	70
Liver-disorder	91	21	91	29.6	29.8
Sonar	793	90	793	104.8	129.2
